# Bundle Branch Reentrant Ventricular Tachycardia

**Published:** 2005-04-01

**Authors:** Alexander Mazur, Jairo Kusniec, Boris Strasberg

**Affiliations:** Cardiology Department, Rabin Medical Center, Beilinson Campus, Petah-Tikva and Sackler School of Medicine, Tel Aviv University, Israel

## Abstract

Bundle branch reentrant (BBR) tachycardia is an uncommon form of ventricular tachycardia (VT) incorporating both bundle branches into the reentry circuit. The arrhythmia is usually seen in patients with an acquired heart disease and significant conduction system impairment, although patients with structurally normal heart have been described. Surface ECG in sinus rhythm (SR) characteristically shows intraventricular conduction defects. Patients typically present with presyncope, syncope or sudden death because of VT with fast rates frequently above 200 beats per minute. The QRS morphology during VT is a typical bundle branch block pattern, usually left bundle branch block, and may be identical to that in SR. Prolonged His-ventricular (H-V) interval in SR is found in the majority of patients with BBR VT, although some patients may have the H-V interval within normal limits. The diagnosis of BBR VT is based on electrophysiological findings and pacing maneuvers that prove participation of the His- Purkinje system in the tachycardia mechanism. Radiofrequency catheter ablation of a bundle branch can cure BBR VT and is currently regarded as the first line therapy. The technique of choice is ablation of the right bundle. The reported incidence of clinically significant conduction system impairment requiring implantation of a permanent pacemaker varies from 0% to 30%. Long-term outcome depends on the underlying cardiac disease. Patients with poor systolic left ventricular function are at risk of sudden death or death from progressive heart failure despite successful BBR VT ablation and should be considered for an implantable cardiovertor-defibrillator.

## Introduction

Bundle branch reentrant (BBR) tachycardia is a form of ventricular tachycardia (VT) incorporating both bundle branches into the reentry circuit. Another variant of the His-Purkinje macro-reentry utilizing ramifications of the left bundle branch is referred to as interfascicular reentrant tachycardia.

Reentry within the His-Purkinje system (HPS) in humans was first documented by Akhtar et al [1,2] in studies involving isolated ventricular beats commonly produced by programmed ventricular stimulation (also known as “V3 phenomenon”). These isolated reentrant beats within the HPS represent a normal response to stimulation. It is well recognized that sustained bundle branch reentry can not be induced in patients with normal HPS. The latter may be explained by electrophysiological properties of normal HPS characterized by the combination of very fast conduction velocity and a relatively long refractory period which precludes formation of a stable reentry circuit. Persistent bundle branch reentry as a mechanism of sustained VT has been demonstrated in patients with conduction system impairment [3-21]. In series of consecutive patients undergoing electrophysiological studies, this mechanism has been responsible for up to 6% of induced sustained monomorphic VT [7-10]. However, there is significant variability in the reported incidence of inducible BBR VT that could be related to the patient population studied as well as to the stimulation protocol used [22].

## Clinical characteristics of patients

BBR VT is usually seen in patients with an acquired structural heart disease and significant conduction system impairment [7-11]. Dilated cardiomyopathy, both ischemic and non-ischemic, is an important underlying cardiac pathology accounting for the majority of cases in the largest reported series [7-10]. The relative contribution of BBR mechanism to inducible sustained monomorphic VT is significantly higher in non-ischemic (up to 40%) than in ischemic etiology (up to 6%) [7-10]. Whether this relates to specific cardiac pathology or to significantly higher total incidence of inducible sustained monomorphic VT in patients with ischemic cardiomyopathy due to much more readily inducible intra-myocardial reentry is not clear. Valvular disease is another relatively common cause of bundle branch reentry. Aortic or mitral valve surgery can facilitate the development of the arrhythmia [11]. Close anatomical proximity of the HPS to the valvular annuli makes it vulnerable to surgical manipulations in this area. In contrast to postoperative intra-myocardial reentrant VT, BBR VT usually occurs in the early postoperative period (within first 2 weeks) and can be associated with preserved systolic left ventricular function. Bundle branch reentry is an important mechanism of arrhythmia in patients with myotonic myocardial dystrophy [12]. The disease is characterized by relatively selective and significant conduction system impairment. In the majority of these patients there is either minor myocardial involvement or none at all. Conduction abnormalities due to sodium channel blockade with flecainide have been implicated in the development of bundle branch reentry [13-14]. Isolated cases of this arrhythmia mechanism have been described in other diseases associated with conduction impairment [15-17]. The arrhythmia has also been reported in patients with idiopathic isolated conduction system disease and no apparent structural heart abnormalities [18-21]

## Baseline ECG

Surface ECG in sinus rhythm characteristically shows intraventricular conduction defects with or without PR interval prolongation. The conduction defects are presented by non-specific or typical bundle branch block patterns with prolonged QRS duration[7-12]. Although total interruption of conduction in one of the bundle branches would theoretically prevent occurrence of bundle branch reentry, an ECG pattern of “complete” bundle branch block may not be an accurate marker of complete conduction block [8-23]. Rarely, BBR VT can also occur in patients with relatively narrow baseline QRS complex suggesting a role of functional conduction delay in the genesis of bundle branch reentry [24].

## Clinical features

**Figure 1:**
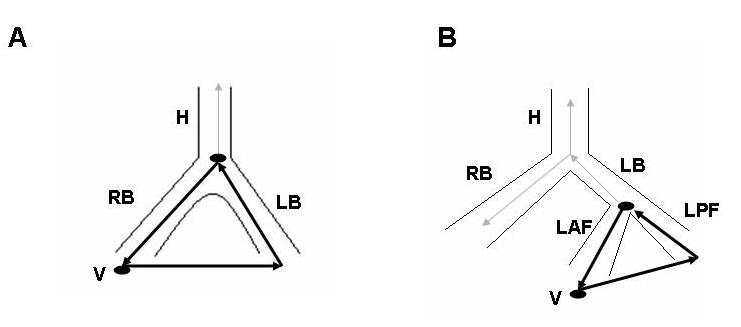
Scheme of bundle branch (**A**) and interfascicular (**B**) reentry. (**A**) Antegrade conduction is over the right bundle (RB) and retrograde conduction is up the left bundle (LB). Because ventricular activation (V) starts at the right ventricular apex, the QRS morphology is a typical LBBB. Reversal of the reentry circuit with activation of the ventricles through the left bundle system is associated with a typical RBBB QRS morphology. Note that retrograde activation of the His bundle (H) occurs during antegrade activation of the RB. See text for discussion. (**B**) Antegrade conduction proceeds over the anterior fascicle (LAF) and retrograde conduction up the posterior fascicle (LPF). Because ventricles are activated through the left bundle system, the QRS morphology has a RBBB pattern. In this type of the circuit, activation of the ventricles occurs over the LAF and is associated with a left anterior fascicular block pattern. Reversal of the reentry circuit with activation of the ventricles over the LPF shows a left posterior fascicular block pattern. Note that the LB and H are activated retrogradely during antegrade activation of the LAF. See text for discussion.

Patients usually present with presyncope, syncope or sudden death because of VT with fast rates frequently above 200 beats per minute [7-12]. The surface ECG QRS morphology during VT is a typical bundle branch block pattern and may be identical to that in sinus rhythm [25]. A left bundle branch block (LBBB) pattern is the most commonly reported VT morphology [7].[12]. In contrast to VT of myocardial origin, bundle branch reentry with a LBBB pattern characteristically shows rapid intrinsicoid deflection in the right precordial leads indicating that initial ventricular activation occurs through the HPS. During BBR VT with a LBBB pattern the activation propagates in the antegrade direction down the right bundle (RB) and in the retrograde direction up the left bundle (LB). During BBR VT with a right bundle branch block (RBBB) pattern the direction of activation is reversed (Figure 1A). The orientation of frontal plane QRS axis is usually to the left. Less frequently it may be to the right or normal. This may relate to the activation pattern of the left bundle branch fascicles.

## Electrophysiological data

Prolonged His-ventricular (H-V) interval in sinus rhythm is found in the majority of patients with BBR VT [7-12]. Although some patients may have the H-V interval in sinus rhythm within normal limits, functional HPS impairment in these patients manifested as H-V interval prolongation or split His-bundle potentials, commonly become evident during atrial programmed stimulation or burst pacing [24].

BBR VT is usually inducible with conventional pacing protocols. Both atrial and ventricular programmed stimulation or burst pacing can be useful. In some patients, the arrhythmia may be inducible only with atrial pacing stressing the importance of using atrial stimulation for evaluation of patients with VT or syncope [19]. Left ventricular stimulation, introduction of short-long-short sequences, isoproterenol infusion, or sodium channel blockade can aid in the induction of bundle branch reentry [7-9]. Atrial fibrillation or flutter may also facilitate the initiation of BBR VT [26]. Most induced BBR VTs demonstrate LBBB morphology. The initiation of bundle branch reentry with RBBB morphology may require left ventricular pacing [18].

**Figure 2:**
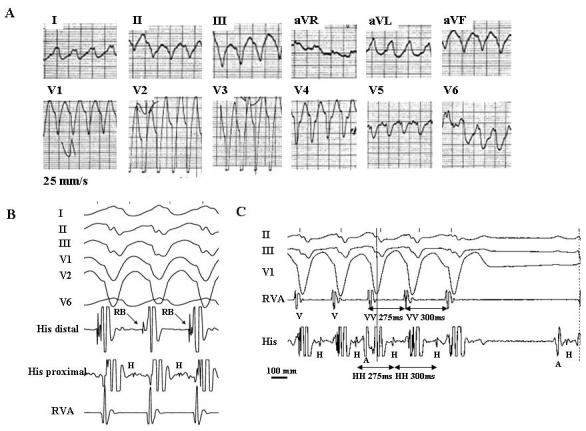
Twelve-lead ECG during a spontaneous episode of bundle branch reentrant tachycardia (**A**). Surface ECG leads I, II, III, V1, V2, V6 (**B**) or II, III, V1 (**C**) and intracardiac recordings from the His bundle (His) and right ventricular apex (RVA) during bundle branch reentrant tachycardia induced in the same patient. The recordings show many characteristic diagnostic features of bundle branch reentrant tachycardia: (1) typical LBBB morphology and left superior axis (**A**); (2) AV dissociation (**C**); (3) H preceding every V with the HV interval (112 ms) greater than that recorded during sinus rhythm (68 ms) (**B and C**); (5) H precedes the right bundle deflection. This sequence is consistent with ventricular activation through the right bundle branch and is appropriate for a LBBB morphology of tachycardia (**B**); (6) spontaneous changes in the HH intervals preceded similar changes in the VV intervals (**C**); and (7) spontaneous termination of tachycardia with retrograde conduction block to H (**C**). A, H, RB, and V denote atrial, His bundle, right bundle, and ventricular electrograms, respectively. (From: Mazur A, Iakobishvili Z, Kusniec J, Strasberg B. Bundle branch reentrant ventricular tachycardia in a patient with the Brugada electrocardiographic pattern. A.N.E. 2003;8:252-255, with permission of Blackwell Futura Publishing, Inc.)

The following electrophysiological features are consistent with the bundle branch reentry mechanism (Figure 2): (1) reproducible initiation of tachycardia with critical V-H interval prolongation suggesting that induction of the tachycardia depends on conduction delay within the HPS; (2) a stable His or bundle branch potential preceding each ventricular activation. The H-V interval is usually longer than that recorded in sinus rhythm. However, in rare cases, it can be equal to or slightly shorter (by less than 15 ms) than the HV interval in sinus rhythm. During bundle branch reentry the His bundle is activated in the retrograde direction simultaneously with the proximal part of the bundle branch serving as the antegrade limb of the reentry circuit (Figure 1A). The relative duration of the H-V interval recorded during VT as compared to sinus rhythm would depend on 2 factors: (a) the balance between antegrade and retrograde conduction times from the upper turnaround point of the reentry circuit; and (b) the site of His bundle recording relative to the upper turnaround point [27]. Conduction delay in the bundle branch used as the antegrade limb of the circuit would tend to prolong H-V interval during VT, while retrograde conduction delay to the His bundle recording site as well as the use of relatively proximal His bundle recording site (far from the turnaround point) would tend to shorten it. The RB (LB)-V interval must always be = than that recorded in sinus rhythm emphasizing the importance of recording the RB (LB) potential during VT [28]; (3) H-RB (LB)-V activation sequence consistent with ventricular activation through an appropriate bundle branch with regard to VT QRS morphology; (4) changes in H-H (RB-RB or LB-LB) interval during VT precede changes in the VV interval. In other words, the tachycardia cycle length is affected by variation in the V-H (V-RB or V-LB) interval. This would strongly argue against passive retrograde HPS activation. However, the opposite relation may not rule out the bundle branch reentry mechanism. V-V oscillations preceding H-H oscillations during BBR VT suggesting conduction variation in the antegrade rather than in the retrograde limb of the reentry circuit have been reported [29,30]. On the other hand, changes in the relationship between V and H (RB or LB) that do not affect the tachycardia cycle length would support lack of participation of the HPS in the tachycardia mechanism; (5) reproducible termination of VT with block in the HPS. This is another finding suggesting that the HPS is not a passive bystander; (6) inability to induce VT after ablation of the right or left bundle branch.

Recording from both sides of the septum may help in the identification of the bundle branch reentry mechanism. Documentation of typical H-RB-V-LB (during VT with LBBB morphology) or H-LB-V-RB (during VT with RBBB morphology) activation sequence would further support BBR VT diagnosis. In addition, during VT with LBBB morphology right ventricular excitation must precede the left ventricular excitation. The opposite is true for the VT with RBBB morphology [22].

Pacing maneuvers, if feasible, can be extremely helpful. Ability to dissociate His or, particularly, RB (LB) potential would strongly argue against bundle branch reentry mechanism. The combination of concealed entrainment (concealed QRS fusion) by atrial pacing and manifest entrainment (manifest QRS fusion) by ventricular pacing has been recently proposed as a useful diagnostic criterion for BBR VT with LBBB QRS morphology [30]. Analysis of the difference between the first postpacing interval after VT entrainment from the right ventricular apex and the tachycardia cycle length can be used to rapidly screen for VT mechanism. Bundle branch reentry is unlikely if the difference is longer than 30 ms [31]. The latter two findings can be potentially helpful in identifying the bundle branch reentry mechanism when stable recording of the His (RB or LB) potential during VT is not possible, although their ultimate utility needs further validation. Application of the pacing maneuvers during bundle branch reentry is often hampered by fast VT rates commonly associated with hemodynamic compromise. Furthermore, entrainment of BBR VT by atrial pacing has a limited success and usually requires isoproterenol infusion to improve atrio-ventricular (AV) nodal conduction.

## Interfascicular tachycardia

**Figure 3:**
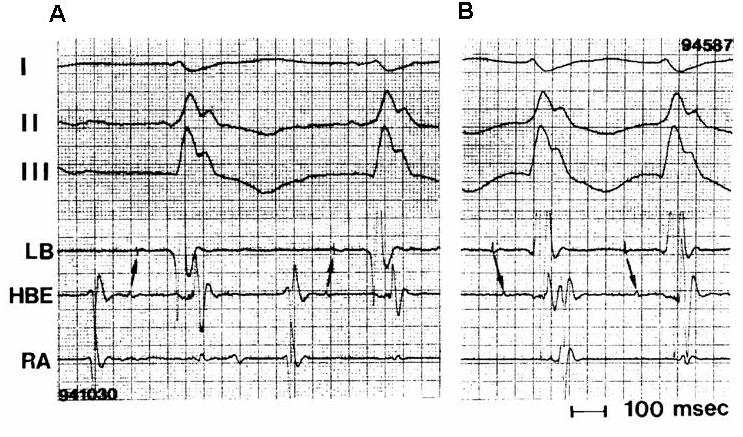
Surface ECG leads I, II, III, and intracardiac recordings from the left bundle (LB), His bundle (HBE), and right atrium (RA) during sinus rhythm (**A**) and interfascicular reentrant tachycardia (**B**). Note characteristic features of interfascicular reentry: (1) the H-V interval during tachycardia (70 ms) is shorter than that in sinus rhythm (100 ms); and (2) the LB potential precedes the His bundle potential. See text for discussion. (From: Crijns HJ, Smeets JL, Rodriguez LM, et al. Cure of interfascicular reentrant ventricular tachycardia by ablation of the anterior fascicle of the left bundle branch. J Cardiovasc Electrophysiol 1995;6:486-492, with permission of Blackwell Futura Publishing, Inc.)

Interfascicular tachycardia has been less commonly reported [9,19,28,32-34]. BBR and interfascicular tachycardia may be present in the same patient [9,19,33,34]. The tachycardia usually has RBBB morphology. The orientation of the frontal plain axis is variable and may depend on the direction of the reentrant circuit. Antegrade activation over the left anterior fascicle and retrograde through the posterior fascicle would be associated with right axis deviation while the reversed activation sequence with left axis deviation. In contrast to BBR VT, the H-V interval during interfascicular tachycardia is usually shorter by more than 40 ms than that recorded in sinus rhythm [22]. This is because the upper turnaround point of the circuit (the left bundle branching point) is relatively far from the retrogradely activated His bundle (Figure 1B). During interfascicular tachycardia the LB potential should be inscribed before the His potential (Figure 3) [22]. During BBR VT with RBBB morphology the His potential usually precedes the LB potential, although the reverse is theoretically possible if retrograde conduction time to the His bundle recording point is significantly prolonged.

## Differential diagnosis

The differential diagnosis of BBR VT includes other mechanisms of VT and different types of supraventricular tachycardia with aberrant conduction. All electrophysiological findings and pacing maneuvers described above that prove participation of the HPS in the tachycardia mechanism and exclude passive retrograde activation of the HPS help to differentiate between bundle branch reentry and other mechanisms of VT. The exclusion of supraventricular tachycardia is particularly important because QRS morphology during BBR VT is a typical bundle branch block pattern and also may be similar to that in sinus rhythm. The differential diagnosis should be based on the complimentary use of the diagnostic criteria of bundle branch reentry as well as supraventricular tachycardia [22]. Since AV dissociation is typically present during BBR VT, the differential diagnosis is usually narrowed to AV nodal reentrant tachycardia, junctional tachycardia and hypothetical mechanisms such as intrahisian reentry and orthodromic tachycardia using a retrograde nodo-fascicular (ventricular) pathway. Orthodromic AV reciprocal tachycardia, tachycardia using an antegrade atrio-fascicular (ventricular) accessory pathway, and atrial tachycardia need to be also considered when 1:1 ventriculo-atrial conduction is present. Entrainment with manifest QRS fusion during ventricular pacing and ability to terminate or reset the tachycardia with ventricular extrastimulus introduced when His bundle is refractory will rule out atrial tachycardia, AV nodal reentrant tachycardia, junctional tachycardia and intrahisian reentry. In all types of supraventricular tachycardia, the difference between the first postpacing interval after entrainment from the right ventricular apex and the tachycardia cycle length should be much longer than 30 ms [31].

## Treatment

Pharmacologic antiarrhythmic therapy, both empiric and electrophysiologically guided, is usually ineffective. Radiofrequency catheter ablation of a bundle branch can cure BBR VT and is currently regarded as the first line therapy. The technique of choice is ablation of the RB [8-10]. Using this technique, the catheter is initially placed at the His bundle area and then gradually advanced toward anterior-superior ventricular septum with clockwise torque. The RB potential is identified by the following characteristics: (1) a sharp deflection inscribed at least 20 ms later than the His potential; and (2) absence of the atrial electrogram on the same recording. The RB-V interval value of < 30 ms may not be a reliable marker of the RB potential in these patients because of the HPS disease that can cause prolongation of RB-V conduction time [8,10,27]. The reported incidence of clinically significant conduction system impairment requiring implantation of a permanent pacemaker varies from 0% to 30% [8-12,24,34]. Ablation of the LB has been proposed in patients with preexisting LBBB pattern on the baseline ECG to reduce the need for pacing [35]. However, the technique is difficult to perform because of non-discrete anatomy of the LB that usually requires application of multiple lesions. Moreover, the RB ablation seems to be safe in the majority of patients with baseline LBBB pattern [8,10,24].

Ablation of interfascicular tachycardia is guided by fascicular potentials. Successful ablation of the arrhythmia can be performed by targeting either the left anterior or posterior fascicle [9,19,32-34].

## Long-term outcome and management

The available data on long-term outcome of patients with BBR VT treated by catheter ablation come from small retrospective series including predominantly patients with left ventricular dysfunction [7-11,24,27]. These data suggest that the prognosis depends on the underlying cardiac disease. Patients with dilated cardiomyopathy and poor systolic LV function, especially those who have inducible VT other than BBR, are at high risk of non-BBR VT recurrence and sudden death despite successful abolition of bundle branch reentry. Progressive heart failure is a common cause of death in this population of patients [7-11,24,27]. Therefore, most of these patients should be considered for an implantable cardiovertor-defibrillator (ICD) with or without cardiac resynchronization capabilities. Because BBR VT has a limited response to antiarrhythmic drugs and can be an important cause of repetitive ICD therapies, catheter ablation of the arrhythmia should always be considered as an important adjunct to the device therapy. The survival and management strategy of patients with bundle branch reentry and preserved left ventricular function who have no other risk markers for sudden death is less clear. Very limited data suggest that these patients may have a favorable long-term prognosis after successful ablation of bundle branch reentry [10,11,18]. Patients with myotonic dystrophy may need prophylactic permanent pacemaker implantation because of the progressive nature of the conduction system disease [12]. The recurrence of BBR VT after successful ablation would be extremely unlikely [8-10]. Complete RBBB pattern on the ECG appears to be a good marker of long-term success after ablation of the RB. However, it still remains unclear whether or not a routine follow-up electrophysiological study should be undertaken in patients who do not have ICD back-up because of potentially life threatening consequences of recurrent BBR VT.
